# Using Synchrotron Radiation-Based Infrared Microspectroscopy to Reveal Microchemical Structure Characterization: Frost Damaged Wheat *vs.* Normal Wheat

**DOI:** 10.3390/ijms140816706

**Published:** 2013-08-14

**Authors:** Hangshu Xin, Xuewei Zhang, Peiqiang Yu

**Affiliations:** 1 Department of Animal Science and Technology, Tianjin Agricultural University, 22 Jinjin Road, Xiqing District, Tianjin 300384, China; 2 Department of Animal and Poultry Science, College of Agriculture and Bioresources, University of Saskatchewan, 51 Campus Drive, Saskatoon, SK S7N 5A8, Canada

**Keywords:** synchrotron, frost damaged wheat, molecular structural make-up

## Abstract

This study was conducted to compare: (1) protein chemical characteristics, including the amide I and II region, as well as protein secondary structure; and (2) carbohydrate internal structure and functional groups spectral intensities between the frost damaged wheat and normal wheat using synchrotron radiation-based Fourier transform infrared microspectroscopy (SR-FTIRM). Fingerprint regions of specific interest in our study involved protein and carbohydrate functional group band assignments, including protein amide I and II (*ca.* 1774–1475 cm^−1^), structural carbohydrates (SCHO, *ca.* 1498–1176 cm^−1^), cellulosic compounds (CELC, *ca.* 1295–1176 cm^−1^), total carbohydrates (CHO, *ca.* 1191–906 cm^−1^) and non-structural carbohydrates (NSCHO, *ca.* 954–809 cm^−1^). The results showed that frost did cause variations in spectral profiles in wheat grains. Compared with healthy wheat grains, frost damaged wheat had significantly lower (*p* < 0.05) spectral intensities in height and area ratios of amide I to II and almost all the spectral parameters of carbohydrate-related functional groups, including SCHO, CHO and NSCHO. Furthermore, the height ratio of protein amide I to the third peak of CHO and the area ratios of protein amide (amide I + II) to carbohydrate compounds (CHO and SCHO) were also changed (*p* < 0.05) in damaged wheat grains. It was concluded that the SR-FTIR microspectroscopic technique was able to examine inherent molecular structure features at an ultra-spatial resolution (10 × 10 μm) between different wheat grains samples. The structural characterization of wheat was influenced by climate conditions, such as frost damage, and these structural variations might be a major reason for the decreases in nutritive values, nutrients availability and milling and baking quality in wheat grains.

## 1. Introduction

In western Canada, wheat (*Triticum aestivum* L.) plays a significant role in the cereal grain export industry, and it is a main ingredient for human consumption. Wheat is rich in starch content (>60%), resulting in a high level of energy content, which is similar to corn grain (NRC). The AC Barrie wheat is popularly and ideally suited to grow in western Canada. However, in western Canada, frost damage to crops is common, due to cold weather, and a heavy frost may cause more than 50% of wheat damage during late maturation. As clearly demonstrated in previous publications, frost damage had detrimental effects on flour quality and baking performance [1,2]. Therefore, millions of tons of frost damaged wheat grain may not be suitable for human consumption any more.

In earlier studies, we compared nutritional values between normal wheat and frost damaged wheat using different evaluation systems [3,4]. Although frozen wheat had decreased nutritive values and different biodegradation kinetic behaviors to ruminants, its total rumen available N (FN) to available carbohydrate (FCHO) ratio (FN/FCHO) was 25 g/kg, which was within the optimal range (25–32 g N/kg total carbohydrates (CHO) [5,6] for rumen fermentation. These results indicated that frost damaged wheat might have a potential to be utilized by ruminants in feed and livestock industries. However, there was no study on the effect of frost on the inherent structure of frost damaged wheat in the literature.

Synchrotron radiation-based Fourier transform infrared microspectroscopy (SR-FTIRM) has been developed as a rapid, direct and non-destructive technique [7,8]. Compared with a conventional standard globar source, synchrotron light is 100–1000 times brighter, and its microspectroscopy can provide a higher signal-to-noise ratio at high-spatial resolutions within plant cellular dimensions [9–11]. Recently, it has been successfully used as a powerful tool to reveal internal chemical structure changes at a cellular or subcellular dimension among various food/feed and plant/seed tissues [12,13]. Additionally, nutrient utilization, bioavailability and feed quality are highly linked to intrinsic molecular structure [14,15]. Protein amide I and II spectral profiles (e.g., peak intensity, ratio of functional group) are related to protein value [16]. The chemical composition and nutrient availability of frost damaged wheat in ruminants have been clearly obtained [3,4]; however, to our knowledge, no comparison has been made in chemical structure of biopolymer functional groups between normal wheat and frost damaged wheat on a molecular basis.

Consequently, the objectives of this study were to compare: (1) protein structure spectral characteristics, including amide I and II bands, as well as protein secondary structure; and (2) carbohydrate internal structure and functional groups spectral intensities between normal wheat and frost damaged wheat using SR-FTIRM. Our research will provide detailed chemical structural and spectroscopic information of frost damaged wheat grain, which would, in large part, explain why frost had a detrimental impact on its nutritional values and biodegradation behaviors, and this knowledge would be helpful for better and effective utilization of frost damaged wheat grains in the feed industry.

## 2. Results and Discussion

### 2.1. Univariate Molecular Spectral Analysis of Protein Amide and Protein Secondary Structure Profiles in Endosperm Tissue of Normal and Frost Damaged Wheat Grains

**Figure 1:**
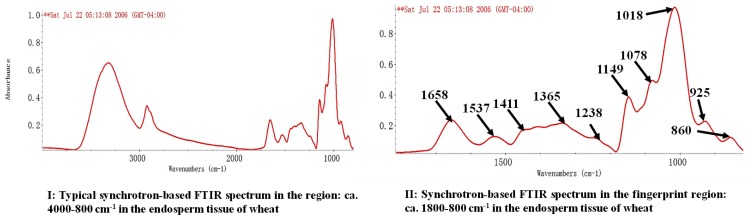

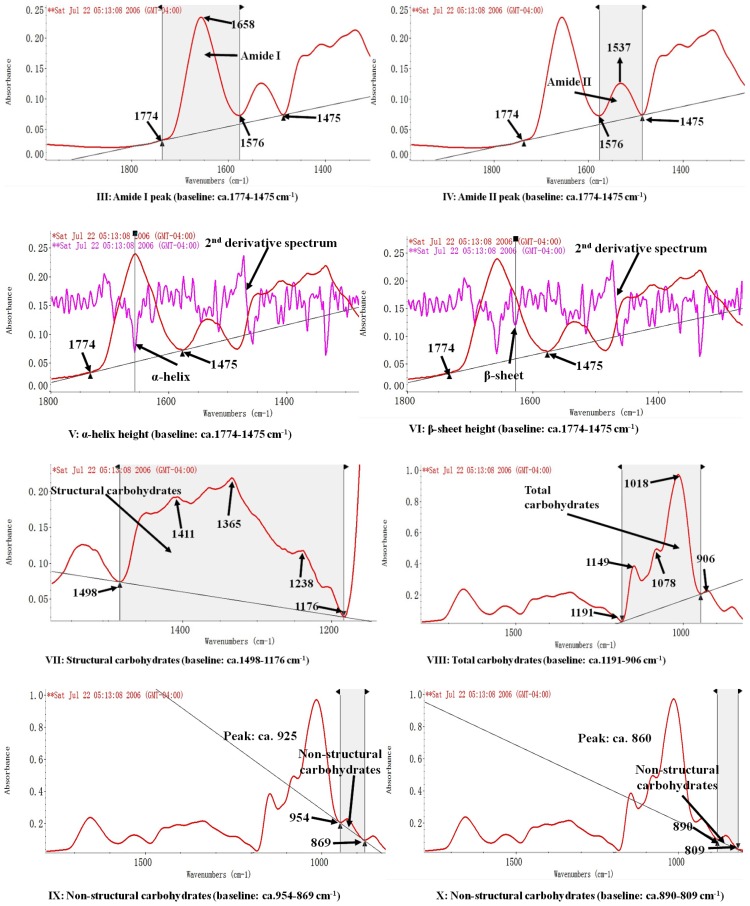
Typical synchrotron-based FTIR spectrum of endosperm tissue within a cellular dimension. (**I**) Whole mid-IR region: *ca.* 4000–800 cm^−1^; (**II**) Fingerprint region: *ca.* 1800–800 cm^−1^; (**III**, **IV**) Amide I and II peak and area; (**V**, **VI**) α-helix and β-sheet heights; (**VII**) Structural carbohydrates area; (**VIII**) Total carbohydrates area; (**IX**, **X**) Non-structural carbohydrates area.

Synchrotron light sourced FTIR microspectroscopy can be used to examine molecular chemistry and intrinsic microstructural characteristics within biological tissues [10,11]. In the current study, the amide I and II peak height and area definition in SR-FTIRM are shown in Figure 1III,IV. Regarding protein secondary structures, like the modeled α-helix and β-sheet, they were identified using the second derivative function in the OMNIC software (Figure 1V,VI). The SR-FTIRM absorbance intensities of protein spectral parameters in the endosperm tissue of two kinds of wheat grains are presented in Table 1. No significant difference could be found between these two wheat samples in protein amide I and II heights and areas. However, as for the spectral ratio profile, the frost damaged wheat significantly reduced (*p <* 0.05) the height and area ratios of amide I to II by 25%–28%, indicating that frost might affect protein intrinsic structural make-up or protein biopolymer conformation of wheat to some extent. The modeled α-helix and β-sheet are the two typical parameters of protein secondary structures [17,18]. In light of our results, frost damaged wheat had similar band absorbance intensities in α-helix and β-sheet heights, as well as their height ratio with normal wheat grains. Several works of research have been published on the protein structural profiles of wheat grains recently [14,16,19], but the results were not in full agreement, which could be explained by different varieties of wheat grains or different spectroscopic techniques used for spectral data collection in these studies. However, the current results were partially consistent with our earlier study [4] in which frost damaged wheat had similar crude protein content (15.9% DM), but different protein sub-fraction profiles (higher contents of rapidly degradable protein sub-fraction and undegradable protein sub-fraction) with normal wheat grains. Furthermore, Preston *et al*. [20] found that frost damage could not cause any change in wheat protein content at the later-maturate stage.

### 2.2. Univariate Molecular Spectral Analysis of Carbohydrate Conformation Profile in Endosperm Tissue of Normal and Frost Damaged Wheat Samples

Table 2 shows the spectral features of structural carbohydrates (SCHO), cellulosic compounds (CELC), total carbohydrates (CHO) and non-structural carbohydrates (NSCHO) from spot samples in the endosperm tissue within cellular dimensions; the comparison between undamaged and frost damaged wheat is revealed using SR-FTIRM. Figure 1(VII)–(X) show typical SR-FTIRM spectra in individual carbohydrate functional groups in wheat grains.

When comparing frost damaged wheat with healthy wheat in the SCHO spectral region, the results showed that frost did reduce (*p <* 0.05) peak heights of the SCHO first peak (0.11 *vs*. 0.09; IR unit) and second peak (0.13 *vs*. 0.11; IR unit), as well as SCHO peak area (26.19 *vs*. 22.19; IR unit). The SCHO region is associated with hemicellulosic and cellulosic compounds [9,21]. Therefore, our results implied that frost damaged wheat might have lower concentrations of hemicellulosic and cellulosic compounds in the endosperm tissue. There were not any differences (*p >* 0.05) in the CELC profile between two wheat samples, with an average of 0.03 IR units and 1.30 IR units in CELC height and area. By analyzing three typical peaks falling in the CHO region (*ca.* 1,191–906 cm^−1^), a similar observation could be found between two kinds of wheat grains. Compared with undamaged wheat, CHO peak heights and areas were notable lower (*p <* 0.05), and CHO area intensity was merely 57.1 IR units in frost damaged wheat. Within the region of *ca.* 954–809 cm^−1^, there were two absorption bands; one peak fell within *ca.* 954–869 cm^−1^ and centered at *ca.* 925 cm^−1^, while the other one fell within 890–809 cm^−1^ and centered at *ca.* 860 cm^−1^. Both bands are assigned to non-structural carbohydrates [15]. Similar to the variation trend in SCHO and CHO spectral profiles, frost damaged wheat had significantly lower IR intensities of NSCHO peak heights and areas, which meant frost could generate a large decrease in the enrichment of NSCHO, like starch in endosperm tissue in wheat kernel. This was in general agreement with the reduced starch volume in frost damaged wheat seed under scanning electron microscopy reported by Cromey *et al*. [22].

The results in our study showed that frost caused large decreases in the contents of SCHO, NSCHO and CHO, which would have the potential to impact nutritive value, food quality and utilization in wheat grains. Additionally, this could explain, in large part, the much lower contents in the non-structural carbohydrates, starch and the rapidly degradable carbohydrate sub-fraction, but the greater enrichment in structural carbohydrates (neutral detergent fibre, acid detergent fibre, and lignin) in the earlier report [4]. The mid-IR spectroscopic information in our study was obtained from spot samples only within the starchy endosperm region and without any consideration on other tissues, like pericarp and seed coat, which were rich in fiber contents. Therefore, this might be responsible for some inconsistencies between our spectral data (Table 2) and chemical analysis results [4] in SCHO measurement. Moreover, compared with the control wheat, frost damaged wheat had significantly different (*p <* 0.05) carbohydrate biopolymer spectral ratios in height ratios of SCHO second to third peaks (1.51 *vs*. 1.78), SCHO first to third peaks (1.25 *vs*. 1.48) and area ratios of SCHO to CHO (0.40 *vs*. 0.33), CELC to CHO (0.03 *vs*. 0.02), as well as SCHO to NSCHO (9.19 *vs*. 7.28). Again, these variations could demonstrate that inherent molecular structural features changed in the endosperm tissue of wheat grains when frost injury occurred.

### 2.3. Univariate Molecular Spectral Analysis of Spectral Ratios of Protein Amide and Individual Carbohydrate Spectral Parameter in Endosperm Tissue of Normal and Frost Damaged Wheat Samples

In order to find out the relative change in spectral absorbance between protein and carbohydrates within the endosperm region in frost damaged wheat grains, some peak height and area ratios associated with these two nutrients were calculated based on corresponding spectral data (Table 3). The results showed that the height ratio of protein amide I (*ca.* 1658 cm^−1^) to CHO third peak (*ca.* 1018 cm^−1^) was 50% higher (*p =* 0.03) in frost damaged wheat than that in undamaged wheat grains. Additionally, a similar tendency (*p =* 0.06) was exhibited in the height ratio of protein amide I to NSCHO first peak (*ca.* 925 cm^−1^) between these two samples. There were also significant differences in the area ratios of protein total amide (amide I + II) to carbohydrate compounds. The area ratios of total amide to CHO and total amide to NSCHO obtained from spot samples in frost damaged wheat were approximately 1.5–1.8-times (*p =* 0.01) those in plump undamaged grains. This phenomenon might result from the large decreases in absorbance intensities within CHO and NSCHO regions and the relatively constant spectral absorbance in the protein amide region (Table 1). As found by Cromey *et al*. [22], who characterized the grain structure of the wheat kernel with a scanning electron microscope, starch granules are mixed with other materials (such as protein matrix) and could not be distinguished in the endosperm tissue of frost damaged wheat in a chemical sense. Additionally, according to another research work [1], more starch granules were fully embedded in protein matrices in damaged wheat endosperm tissue when compared to healthy wheat kernels. Therefore, with the greater association of the protein matrix, more starch in frost damaged wheat could escape from ruminal degradation and, then, be digested in the intestinal tract, which would contribute more for milk synthesis [23]. This was also consistent with nutritive value evaluation in our earlier study [4] in which much higher content of rumen degradable starch was found in plump healthy wheat. Although frost damaged wheat grains had reduced milling and baking quality [1,24] and might not be suitable for human consumption, it could be adopted as an ingredient in animal rations in the livestock industry.

## 3. Experimental Section

### 3.1. Wheat Samples

Identified frost damaged wheat and normal wheat (AC Barrie) used in our study were provided and identified by the Prairie Feed Resource Center (PFRC), University of Saskatchewan, Canada. The normal wheat had plumper kernel than the frost damaged wheat. The detailed sample description and origin were reported in Yu and Racz [3,4].

### 3.2. Advanced Synchrotron Radiation-Based FTIR Microspectroscopy

The spectroscopic experiment was conducted to reveal chemical functional characteristics of wheat and frost damaged wheat using SR-FTIRM at the National Synchrotron Light Source in the Brookhaven National Laboratory (NSLS-BNL, New York, NY, USA) (Beamline scientist, Dr. Lisa Miller). The preliminary study on feed structure study was carried out at the Canadian Light Source (CLS) mid-IR station. Firstly, for each kind of wheat grain, five wheat kernels were randomly selected and, then, cut into thin cross-sections (*ca.* 6 μm thickness) using a microtome at the Western College of Veterinary Medicine at the University of Saskatchewan. Then, the sections were rapidly transferred to BaF_2_ windows (size, 13 mm × 1 mm disk, Spectral System Inc., Hopewell Junction, NY, USA). For each section, approximately 30 spots were randomly selected within the endosperm tissue region between 100 and 600 μm from outside of the seed section [25] and scanned under SR-FTIRM. In this region, the tissues are still heterogeneous. The IR spectra were obtained in the mid-IR range from *ca.* 4000 to 800 cm^−1^, with 256 co-added scans at a resolution of 4 cm^−1^ in the transmittance mode. The pixel size was 10 ×10 μm. A background spectrum was collected from an area free of sample. The baseline correction was done using “auto-baseline corrections” in Process Option in OMNIC software. The assignment of functional group bands were referred to in the literature published previously [15,19].

### 3.3. Univariate Spectral Analysis

Univariate molecular spectral analysis from SR-FTIRM was carried out by using OMNIC 7.2 software (Spectra Tech., Madison, WI, USA). Regions of specific interest in our study involved protein and carbohydrate functional group band assignments. Protein parameters included amides I and II height and area, α-helix and β-sheet peak height and their corresponding spectral ratios. The baseline was *ca.* 1774–1475 cm^−1^ for both protein amide profile and protein secondary profile. Protein amides I and II peaks fell within the range of *ca.* 1662–1641 cm^−1^ and *ca.* 1552–1529 cm^−1^, respectively, and the peaks of modeled α-helix and β-sheet ranged *ca.* 1660–1641 cm^−1^ and *ca.* 1635–1621 cm^−1^, respectively. We also measured spectral parameters that were mainly associated with carbohydrate conformation, including: (1) structural carbohydrates (SCHO, peaks height and area baseline *ca.* 1498–1176 cm^−1^; there were three peaks in this region, with the first, second and third peaks falling within the range of *ca.* 1419–1402, *ca.* 1369–1351 and *ca.* 1245–1230 cm^−1^, respectively), which were highly related to hemi- and cellulosic compounds; (2) cellulosic compounds (CELC, peak height and area baseline *ca.* 1295–1176 cm^−1^ and the peaks falling within the range of *ca.* 1245–1230 cm^−1^); (3) total carbohydrates (CHO, peaks height and area baseline *ca.* 1191–906 cm^−1^; there were three peaks in this region, with the first, second and third peaks falling within the range of *ca.* 1157–1145, *ca.* 1081–1074 and *ca.* 1047–985 cm^−1^, respectively); and (4) non-structural carbohydrates (NSCHO, peaks height and area baseline *ca.* 954–809 cm^−1^; there were three peaks in this region, with the first, second and third peaks falling within the range of *ca.* 965–917 cm^−1^ and *ca.* 925–842 cm^−1^, respectively). Spectral peak intensity height and area ratios were calculated based on relevant spectral data. Figure 1I shows the typical SR-FTIRM spectrum in the region of *ca.* 4000–800 cm^−1^ in the endosperm tissue of wheat grains. The SR-FTIRM spectra of protein and carbohydrate functional groups within the region of *ca.* 1800–800 cm^−1^ are presented in Figure 1II.

### 3.4. Statistical Analysis

Spectral data of normal wheat and frost damaged wheat were statistically analyzed using the Mixed Model procedure of SAS 9.2, and the model was:

(1) $${\text{Y}}_{ijk}=\mu +{\text{T}}_{i}+\text{K}{\left(\text{T}\right)}_{j}+{\text{e}}_{ijk}$$

where Y*_ijk_* was the observation of the dependent variable, *ijk*, μ was the fixed effect of the population mean of the variable, T*_i_* was a fixed effect of treatments (*i* = 2; normal wheat and frost damaged wheat), K(T)*_j_* was the wheat kernels nested within treatments and e*_ijk_* was the random error associated with the observation, *ijk*.

Model assumption checking was carried out by Residual Analysis using Proc Univeriate with Normal and Plot options. Statistical significance was declared and detected at *p <* 0.05, while trends were declared at *p* ≤ 0.10.

## 4. Conclusions

The inherent molecular structure features in endosperm tissue could be examined by using synchrotron-based FTIRM technique at ultra-spatial resolution between different types of wheat grains. Frost did cause variations in spectral profiles in wheat grains. Compared with healthy wheat seed, frost damaged wheat had lower height and area ratios of protein amide I to II and significantly lower absorbance values in almost all the spectral parameters of carbohydrate-related functional groups, including structural carbohydrates, total carbohydrates and non-structural carbohydrates. Furthermore, the height ratio of protein amide I to the CHO third peak and the area ratios of protein amide (amide I + II) to carbohydrate compounds (CHO and NSCHO) were changed in damaged wheat kernels. In conclusion, the structural characterization of wheat could be influenced by climate conditions, such as frost damage. These variations might be a major reason for the decreases in nutritive value, nutrients availability and milling and baking quality in frost damaged wheat grains. In future studies, both the SEM technique and SR-FTIRM technique can be used together to study the effect of climate on the changes of seed inherent structure in both the physical and chemical sense.

## Figures and Tables

**Table 1:** Structural characteristics of protein amide profile and protein secondary structure profile in the endosperm tissue of wheat samples, revealed using synchrotron-based FTIR microspectroscopy: normal wheat compared with frost damaged wheat.

	Baseline (cm^−1^)	Peak (cm^−1^)	Normal wheat	Frost damaged wheat	SEM c	*p*
Protein amides profile a						
Amide I height	1774–1475	~1658	0.13	0.11	0.012	0.50
Amide II height	1774–1475	~1537	0.04	0.05	0.004	0.28
Height ratio of amide I to II	1774–1475		3.34 a	2.50 b	0.173	0.01
Amide I area	1774–1475	~1658	8.81	8.77	0.910	0.97
Amide II area	1774–1475	~1537	2.13	2.77	0.254	0.11
Total amide (amide I + II) area	1774–1475		10.94	11.54	1.128	0.72
Area ratio of amide I to II	1774–1475		4.56 a	3.28 b	0.353	0.03

Protein 2nd structure profile b						
α-helix	1774–1475	~1658	0.12	0.11	0.012	0.55
β-sheet	1774–1475	~1627	0.09	0.08	0.007	0.74
Height ratio of α-helix to β-sheet	1774–1475		1.43	1.36	0.041	0.21

a Protein amide data unit, IR absorbance unit; protein amide I region, 1774–1576 cm^−1^; protein amide II region, 1576–1475 cm^−1^.

b The peaks of α-helix and β-sheet fell within the range of *ca.* 1660–1641 cm^−1^ and *ca.* 1635–1621 cm^−1^, respectively.

c SEM, standard error of the mean. Means with the different letters in the same row are significantly different (*p <* 0.05).

**Table 2:** Structural characteristics of carbohydrate conformation in the endosperm tissue of wheat samples, revealed using synchrotron-based FTIR microspectroscopy: normal wheat compared with frost damaged wheat.

	Baseline (cm^−1^)	Peak (cm^−1^)	Normal wheat	Frost damaged wheat	SEM f	*p*
Structural carbohydrates (SCHO) profile a						
SCHO peak 1 height	1498–1176	~1411	0.11 a	0.09 b	0.006	0.03
SCHO peak 2 height	1498–1176	~1365	0.13 a	0.11 b	0.007	0.03
SCHO peak 3 height	1498–1176	~1238	0.08	0.07	0.002	0.10
SCHO area	1498–1176		26.19 a	22.19 b	1.168	0.04

Cellulosic compounds (CELC) profile b						
CELC height	1295–1176	~1236	0.03	0.03	0.001	0.25
CELC area	1295–1176	~1236	1.32	1.28	0.060	0.60

Total carbohydrates (CHO) profile c						
CHO peak 1 height	1191–906	~1149	0.34 a	0.24 b	0.026	0.02
CHO peak 2 height	1191–906	~1078	0.45 a	0.30 b	0.037	0.02
CHO peak 3 height	1191–906	~1018	0.79 a	0.50 b	0.070	0.02
CHO peak1 area	1191–906	~1149	13.53 a	9.41 b	1.024	0.02
CHO peak 2 area	1191–906	~1078	15.80 a	10.57 b	1.256	0.02
CHO peak 3 area	1191–906	~1018	53.23 a	37.06 b	4.099	0.02
Total CHO area	1191–906		82.56 a	57.05 b	6.364	0.02

Non-structural carbohydrates (NSCHO) profile d						
NSCHO peak 1 height	954–869	~925	0.06 a	0.04 b	0.004	0.01
NSCHO peak 2 height	890–809	~860	0.09	0.05	0.018	0.18
NSCHO peak 1 area	954–869	~925	2.01 a	1.37 b	0.176	0.03
NSCHO peak 2 area	890–809	~860	2.01 a	1.16 b	0.161	0.0002
Total NSCHO area	954–809		3.69 a	2.44 b	0.277	0.01

Spectral ratio profile e						
Height ratio of SCHO peak 1 : 2	1498–1176		0.83	0.83	0.013	0.90
Height ratio of SCHO peak 2 : 3	1498–1176		1.78 a	1.51 b	0.062	0.01
Height ratio of SCHO peak 1 : 3	1498–1176		1.48 a	1.25^b^	0.057	0.02
Height ratio of CHO peak 1 : 2	1191–906		0.81	0.80	0.040	0.89
Height ratio of CHO peak 2 : 3	1191–906		0.58	0.60	0.011	0.18
Height ratio of CHO peak 1 : 3	1191–906		0.44	0.48	0.014	0.08
Area ratio of SCHO : CELC	1498–1176/1295–1176		23.41	17.63	2.506	0.14
Area ratio of SCHO : CHO	1498–1176/1191–906		0.33 b	0.40 a	0.019	0.02
Area ratio of CELC : CHO	1295–1176/1191–906		0.02 b	0.03 a	0.002	0.01
Area ratio of CHO peak 1 : 2	1191–906		0.86	0.89	0.011	0.09
Area ratio of CHO peak 2 : 3	1191–906		0.30	0.28	0.004	0.054
Area ratio of CHO peak 1 : 3	1191–906		0.25	0.25	0.004	0.68
Area ratio of SCHO : NSCHO	1498–1176/954–809		7.28 b	9.19 a	0.338	0.004
Area ratio of CHO : NSCHO	1191–906/954–809		22.27	23.52	0.726	0.26
Area ratio of CELC : NSCHO	1295–1176/954–809		0.69	0.62	0.167	0.77

a Carbohydrate data unit, IR absorbance unit; there were three peaks in this region, and the first, second and third peak regions were *ca.* 1419–1402, *ca.* 1369–1351 and *ca.* 1245–1230 cm^−1^, respectively.

b The cellulosic compounds (CELC) peak region was *ca.* 1245–1230 cm^−1^.

c There were three peaks in this region, and the first, second and third peak regions were *ca.* 1157–1145, *ca.* 1081–1074 and *ca.* 1047–985 cm^−1^, respectively.

d There were two peaks in the non-structural carbohydrate (NSCHO) region, and the first and second peak regions were *ca.* 965–917 cm^−1^ and *ca.* 925–842 cm^−1^, respectively.

e Different spectral ratios were calculated on the basis of relevant data.

f SEM, standard error of the mean. Means with the different letters in the same row are significantly different (*p <* 0.05).

**Table 3:** Structural characteristics of the ratios of protein amide and structural carbohydrates, cellulosic compounds, total carbohydrates and non-structural carbohydrates in the endosperm tissue of wheat samples, revealed using synchrotron-based FTIR microspectroscopy: normal wheat compared with frost damaged wheat.

	Baseline (cm^−1^)	Peak (cm^−1^)	Normal wheat	Frost damaged wheat	SEM a	*p*
Height ratio of amide I : CEL	1774–1475/1295–1176	~1658/~1236	4.63	4.80	0.457	0.81
Height ratio of amide I : CHO peak 3	1774–1475/1191–906	~1658/~1018	0.18 ^b^	0.27 a	0.023	0.03
Height ratio of amide I : NSCHO peak 1	1774–1475/954–869	~1658/~925	2.20	3.32	0.367	0.06
Height ratio of amide I : NSCHO peak 2	1774–1475/890–809	~1658/~860	2.65	3.22	0.331	0.25
Area ratio of total amide : SCHO	1774–1475/1498–1176		0.54	0.65	0.102	0.47
Area ratio of total amide : CEL	1774–1475/1295–1176		11.81	10.46	2.647	0.73
Area ratio of total amide : CHO	1774–1475/1191–906		0.15 ^b^	0.23 a	0.018	0.01
Area ratio of total amide : NSCHO	1774–1475/954–809		3.22 ^b^	5.66 a	0.513	0.01

a SEM, standard error of the mean. Means with the different letters in the same row are significantly different (*p* < 0.05).
